# Cutis Laxa syndrome: a case report

**DOI:** 10.11604/pamj.2015.20.3.5878

**Published:** 2015-01-05

**Authors:** Mohamed Hbibi, Sana Abourazzak, Mounia Idrissi, Sana Chaouki, Samir Atmani, Moustapha Hida

**Affiliations:** 1 Department of Pediatrics, University Hospital Hassan II Fès, Morocco

**Keywords:** Cutis laxa, connective tissue, emphysema

## Abstract

Cutis laxa (CL) is a heterogeneous group of inherited and acquired connective tissue disorders characterized by a loose skin and variable systemic involvement (inguinal hernia, cardiopulmonary disease, and emphysema). Autosomal dominant, autosomal recessive and x-linked recessive patterns have been described in the inherited forms. Acquired forms of this disease have been associated with a previous inflammatory skin disorder (urticaria…). The characteristic symptomatological pattern is resulting from paucity of elastic fibers. We report an 18 months old baby boy with a congenital cutis laxa. He was admitted in pediatric unit for respiratory disorders. The diagnosis of CL syndrome is based on clinical assessment of typical skin features and the associated extracutaneous finding.

## Introduction

Cutis laxa is a rare disorder of unknown cause characterized by progressive looseness of the skin associated with abnormalities of others organs and structures containing elastic tissue such as lung, vasculature, or gastrointestinal tract [1]. Both inherited and acquired forms exist. The most clinical feature is loose and pendulous skin, sagging of cheeks, or prematurely aged appearance. The disease is characterized by a generalized reduction in the amount and size of elastic fibers and fragmentation and disruption of their normal arrangement [2, 3].

## Patient and observation

**Figure 1:**
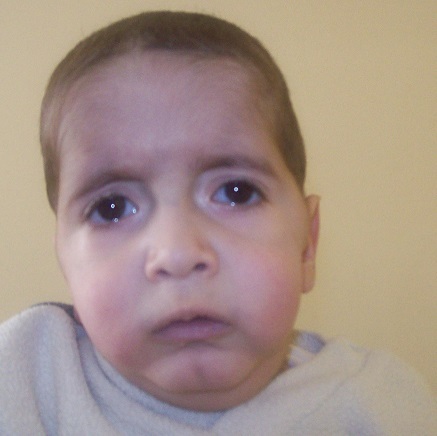
Senile appearance of the face

**Figure 2:**
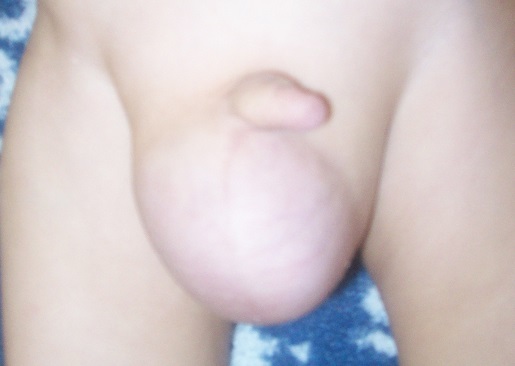
Right-sided inguinal hernia

Z.Z, an 18 months old boy, the child of related parents, presented with respiratory disorders associated with the fever and cough. There was a history of recurrent episodes of cough and breathlessness since age of 4 months, with an increase in the frequency and severity of the episodes in the few past weeks. He was receiving treatment from his practitioner, but was referred for increasing breathlessness. Early development was normal, except language and dentition delayed. There was no family history. On examination, the child was febrile and had respiratory distress with intercostal and subcostal retractions. The respiratory rate was 60/min, heart rate 110/min, and blood pressure 100/70 mmhg, SaO_2_ was 60% in the ambient air and then increased up to 94% using 2l of oxygen. The weight and height were 10 kg (under 2DS) and 70 cm (normal for that age) respectively. The face had a senile appearance with pendulous ear lobes and lax skin (Figure 1). His thorax is deformed with scoliosis. There was a right-sided, reducible, inguinal hernia (Figure 2). There was no laxity of joints.

Chest examination revealed the bilaterally creptations. Cardiovascular examination was normal with first and second heart sounds were heard normally. Examination of the abdomen was normal with no palpable liver and spleen. Others systems were normal. Test showed hemoglobin of 8.7 g/dl, a total WBC count of 17270/mm^3^ with a differential count of polymorphs of 42%, lymphocytes of 56%. Routine biochemical investigations yielded normal results. CRP was 26 mg/l TORSH (toxoplasmosis, rubella, syphilis, herpes) serologies were negatives. A chest x-ray showed emphysema in the left lung, opacities in the right lung and cadiomegaly with a cardiothoracic ratio of 0.6. Electrocardiography showed aortic dilatation, mitral valve prolapsed and sign of pulmonary arterial hypertension. Thoracic computed tomography revealed alveolar condensations and localizations of emphysema. Gastric fibroscopy was normal The child was treated with bronchodilators and antibiotics.

## Discussion

Cutis laxa is a rare connective tissue disorder characterized clinically by loosely hanging skin folds. Histologically, there are changes in dermal elastic fibers It is generally an inherited condition. The mode of transmission can be autosomal recessive (OMIM 219100) with the severest clinical manifestations [4, 5]. Children with the disease have obviously loose skin at birth, and most die during infancy from cardiopulmonary complications. Although many systems may be affected. It has been shown to be caused by a fibulin 5 mutations [6]. In contrast autosomal dominant (OMIM123700) inheritance is associated with mild condition without systemic abnormalities; this form is caused by mutations in the elastin gene [7]. The third type of cutis laxa is transmitted x-linked inheritance (OMIM 304150), it is also termed occipital horn syndrome which is allelic to menkes disease. In our patient, there was no history of any similar problem in any of the family members, thus ruling out an autosomal dominant inheritance. He had no history of developmental delay nor did he have any joint laxity, our patient probably suffered from type I recessive form. He had the characteristic cutaneous abnormalities described in all the varieties of cutis laxa. Additionally, he had inguinal hernia, pulmonary emphysema, and stenosis of pulmonary arteries. Cardiopulmonary abnormalities are common in type I recessive cutis laxa and are the main factors to determine the prognosis and life expectancy. Pulmonary emphysema, and right-sided heart failure caused by pulmonary disease have been commonly described. Various cardiovascular abnormalities including aortic aneurysm, pulmonary artery stenosis as in our patient have been reported with this form of congenital cutis laxa. Ocular anomalies (ectropion, entropion…) have described in autosomal recessive form [8] but in our patient there were no ophthalmological signs of this disease

## Conclusion

Cutis laxa is a rare disorder characterized by a clinical and genetic polymorphism. Patients with the autosomal recessive type have a high risk of serious cardiopulmonary complications.
